# A rare case of Erdheim-chester disease reported from Nepal

**DOI:** 10.1016/j.amsu.2022.104232

**Published:** 2022-07-31

**Authors:** Sunit Chhetri, Srista Manandhar, Durga Neupane, Sushil Sharma Subedi, Sunny Chhetri, Astha Acharya, Sushant Chaudhary, Pradeep Khatiwada, Suraj Shrestha

**Affiliations:** a BP Koirala Institute of Health Sciences, Ghopa Camp, Dharan-18, Sunsari, Province 1, 56700, Nepal; b Department of Surgery, BP Koirala Institute of Health Sciences, Ghopa Camp, Dharan-18, Sunsari, Province 1, Nepal; c Tribhuvan University Teaching Hospital, Maharajgunj, Kathmandu, Province 3, Nepal

**Keywords:** Case report, Erdheim-chester disease, Non-Langerhans histiocytosis

## Abstract

**Introduction:**

Erdheim-Chester Disease (ECD) is a rare non-Langerhans cell histiocytosis with a propensity to involve multiple organs.

**Case presentation:**

We report a case of a patient in mid-60s with occipital headache and ataxia. Following the radiological and immunohistochemical investigations and genomic studies, a diagnosis of ECD was made with two intracerebral lesions. Brain lesions were resected and the patient was discharged with the medication Vemurafenib. After 3 years of diagnosis and 13 years of initial presentation, patient passed away.

**Discussion:**

ECD frequently presents with Diabetes Insipidus as initial presentation, long bone osteosclerosis as the most common presentation, and has multi-system predisposition. ECD can be differentiated from Langerhans Cell Histiocytosis (LCH) with immunohistochemistry images of the biopsy specimens. Further, with genomic analysis of ECD, the neoplastic nature has been highlighted and targeted therapies like Vemurafenib and Cobimetinib are shown to be effective.

**Conclusion:**

Good clinical judgement and supporting investigations can aid in diagnosing rare entities like ECD even in resource-limited settings.

## Highlights

• Rare case of Erdheim-Chester disease with multi-system involvement presenting with initial features 10 years prior to diagnosis.
• Initially Diabetes Insipidus, followed by long bone and other multi-system involvement important cues for the diagnosis.
• Biopsy of the lesion and immunohistochemical analysis can help confirm the diagnosis.
• Genomic studies with mutational analysis further highlights the neoplastic nature of the disease.
• Advancement of genomic studies with targeted therapies like Vemurafenib and Cobimetinib key in of management of ECD.

## 1 Introduction

Lipoid granulomatosis was first explained by Erdheim and Chester in 1930 [1]. After its discoverers, now known as Erdheim-Chester disease (ECD) is a rare, non-Langerhans histiocytosis characterized by xanthogranulomatous infiltration typically affecting long bones, cardiovascular system, retroperitoneum, and central nervous system (25%–50%) [2].

ECD mostly affects adults (mean age at diagnosis, 55 years) with a male preponderance [3]. In 2006, an introductory study stated the time gap between symptom onset and diagnosis to be several months to 25 years [4]. Recent progress in genomic studies, BRAF mutations (BRAF^V600E^) pattern has pointed to neoplastic nature of disease rather than inflammatory [5].

Herein, we report a case of a patient in mid-60s, with complaints of occipital headache, ataxia and blurring of vision along with the approach to diagnosis and management of ECD and genomic studies of disease revealing the true nature. This case report has been reported in line with the SCARE Criteria [6].

## 2 Presentation of case

A 64-year-old right-handed man, known case of Diabetes Insipidus (since 10 years), Empty Sella Syndrome (since 6 years) and Periorbital Xanthogranuloma (since 3 years) presented with complaints of occipital headache, imbalance in walking, restriction of neck movements and blurring of vision since 1 and half months. Previously he underwent thecoperitoneal shunting for Empty Sella Syndrome and was under regular ddavp nasal spray for Diabetes Insipidus. Family history was insignificant.

On examination his vitals were stable and Glasgow Coma Scale was 15/15 (E4V5M6). His pupil was 3mm in size and bilateral reactive. On CNS examination, his higher mental function was intact, meningeal signs absent, sensory intact, power 5/5 of bilateral upper and lower limbs and gait ataxia was present. His respiratory and cardiovascular examinations were all within normal limits.

Routine blood investigations were within normal limits. MRI scan of the brain was performed which suggested 2 separate lesions in the posterior aspect (Fig. 1a). Larger extra-axial lesion measuring 5.51cm × 4.99cm X 4.84cm in the posterior mid part of tentorium with lobulated projection compressing the posterosuperior part of both cerebellum and displacing 4th ventricle anteriorly. Smaller intradural extramedullary lesion measuring 3.02cm × 2.41cm in the cervico-vertebral junction in the region of foramina magnum compressing and displacing the cord towards the left side and superiorly abutting the medulla oblongata and inferiorly reaching up to the level of the odontoid peg was seen.

**Fig. 1a:**
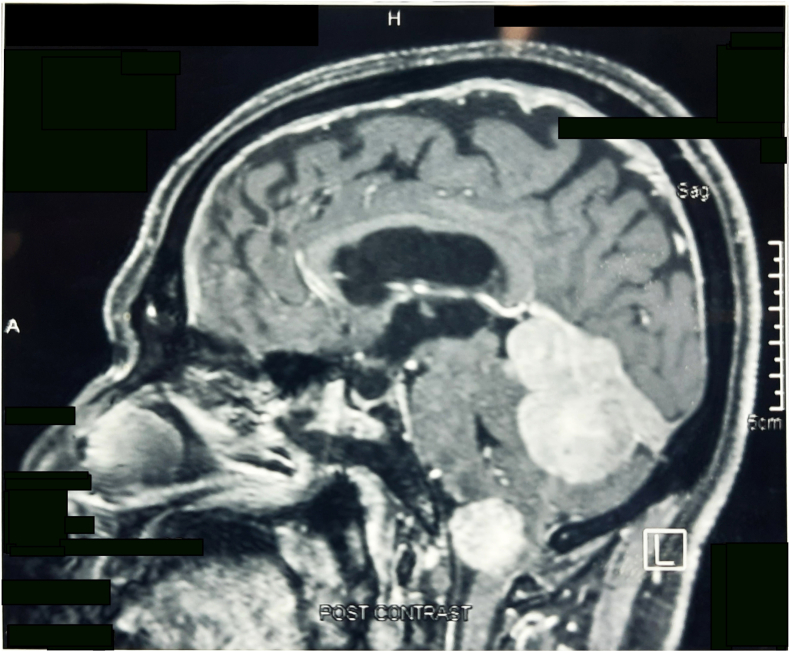
MRI Brain shows two separate extra-axial lesions in the posterior aspect.

^18^F-FDG PET-CECT whole-body scan was done and lesions corresponding to previous brain MRI scan were seen (Fig. 1b). Bilaterally symmetrical osteosclerosis of long bones was also visualized.

**Fig. 1b:**
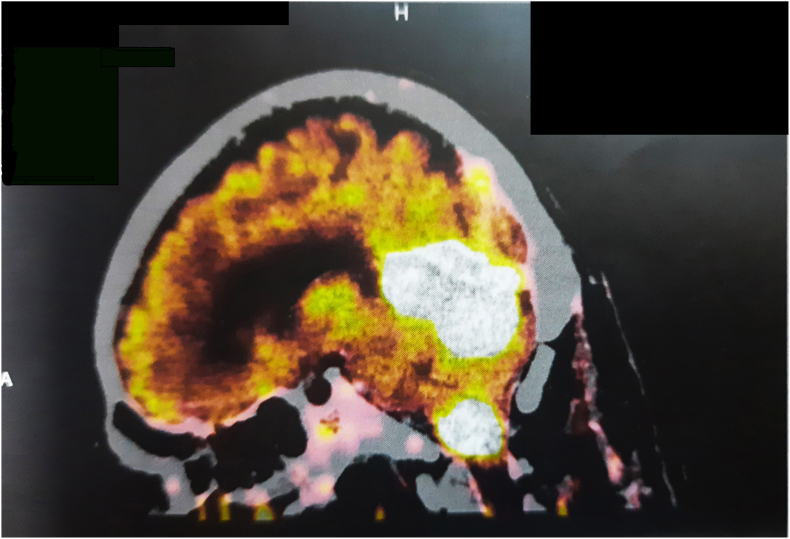
18F-FDG PET-CECT brain shows two discrete FDG avid enhancing masses.

Bone marrow aspiration and biopsy were done. Biopsy revealed lipid-laden histiocytes having foamy eosinophilic cytoplasm with interspersed lymphomonuclear cells with peripheral fibrosis. The histiocytes were CD68 (Leica, clone 514H2) positive and CD1a (Dako, Clone O10)/S100-negative. BRAF V600E (valine to glutamic acid) mutation was also detected. Diagnosis of Erdheim Chester disease was made.

Following confirmation of diagnosis, midline suboccipital craniotomy with C1 laminectomy and subtotal excision of foramen magnum lesion and the tentorial lesion was performed in our setting. Resected brain specimen was sent for the immunohistochemical examination which further consolidated the bone marrow biopsy findings. The patient was discharged with Vemurafenib.

Repeat MRI was performed at 1 month, 6 month and 11-month intervals. The study in comparison with previous MRIs revealed stable findings (Fig. 1c). In due course of time periorbital xanthogranuloma had significantly resolved and patient was content with intervention provided. Only remaining complaint was weakness of the whole body.

**Fig. 1c:**
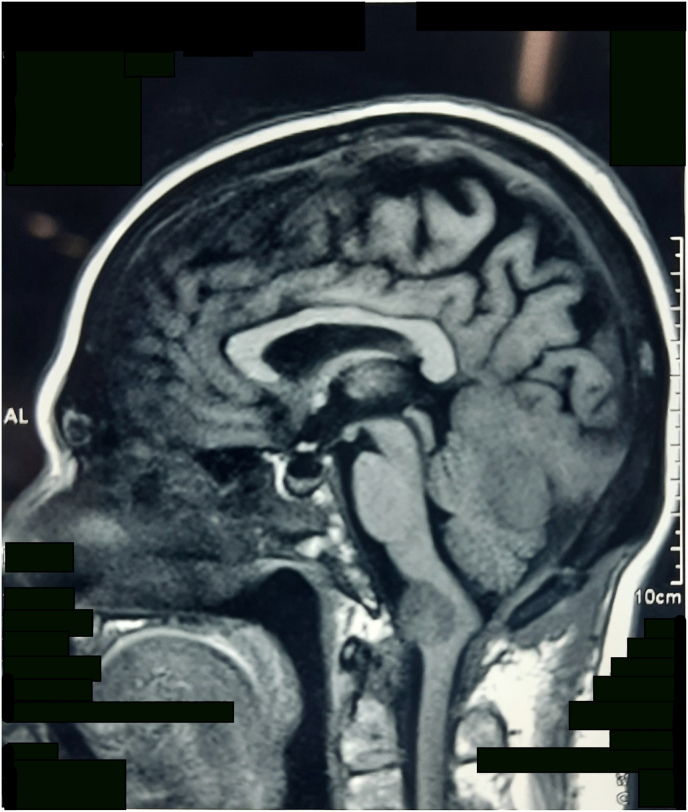
Post-surgery MRI brain shows no significant interval change in the sizes of extra-axial mass adjoining the cerebellar hemisphere and medulla.

In July of 2021, after 3 years of diagnosis and 13 years of initial presentation, the patient passed away with the cause apparently being bleeding and hematoma formation at the site of tumor resection.

## 3 Discussion

This report illustrates the course of the disease and attempts to highlight its true nature.

Initial manifestation observed among patients differs considerably [1]. Diabetes Insipidus of unknown origin is the most frequent first sign to appear and may occur several decades before the diagnosis of ECD [7]. Present in 80–95% of patients, long bone osteosclerosis is one of the most common clinical manifestations of ECD. Whereas in LCH, the osteolytic lesion is found. Periorbital xanthelasma is the most frequent cutaneous manifestation accounting for 22% of patients [3]. Cerebellar (41%) and pyramidal (45%) syndromes are common neurological signs [8].

In consistency with the above clinical findings, the patient first presented a decade back with Diabetes Insipidus, then periorbital xanthelasma and later progressed to have features suggestive of CNS lesion.

With biopsy of affected tissue, diagnosis of ECD and exclusion of LCH is made with the identification of CD68 positive non-Langerhans’ histiocytes with foamy or eosinophilic cytoplasm within polymorphic granuloma or fibrosis. Features of LCH, Birbeck granules, CD1a and S100 positive Langerhans cells are lacking in ECD. With immunohistochemical pictures, ECD and LCH are evidently distinguished [2]. A similar picture was present in the immunohistochemical staining of the biopsy specimen and a conclusive diagnosis of ECD was made.

Since 2010, mutational analysis of ECD has made much needed progress. Almost 54% of ECD and 38% of LCH patients are shown to have a mutation of BRAFV600E initiating the RAS-RAF-MEK-ERK pathway [1]. In line with the aforementioned genetic testing modality, the patient presented with a similar pattern of mutation. This also further highlighted the neoplastic nature of the disease.

Genomic studies of ECD, besides diagnosis, have an even significant role in the management of ECD with targeted therapies like Vemurafenib (BRAF pathway inhibitor) and Cobimetinib (MEK inhibitors) [5]. In support of the efficacy of vemurafenib, the patient improved symptomatically in relation to periorbital xanthelasma which was in a resolving pattern.

The presence of cardiovascular lesions and central nervous system lesions is a poor prognostic factor [1]. The patient struggled with CNS symptoms post-surgery and ultimately expired due to the same further highlighting the poor prognosis.

## 4 Conclusion

Despite the rarity of ECD, clinicians should always be vigilant while dealing with multisystem manifestations presenting with Diabetes insipidus as the initial presentation. This will definitely assist in the early diagnosis and treatment of this debilitating condition and improve prognosis and patient satisfaction to a great extent despite its fatal outcome.

## Ethical approval

Ethical approval for a case report is deemed unnecessary by BPKIHS Institute Review Board and Nepal Health Research Council (NHRC). Consent from the patient is regarded as sufficient for the protection of human subjects.

## Sources of funding

No sources of funding for our research.

## Author contribution

Sunit Chhetri, Srista Manandhar, Durga Neupane; wrote the initial draft of the manuscript. Sushil Sharma Subedi, Sunny Chhetri, Astha Acharya; played a major role in the acquisition of all relevant data. Sushant Chaudhary, Pradeep Khatiwada and Suraj Shrestha; helped in the analysis and interpretation of data and reshaped the manuscript into its final form. The final version of the manuscript was approved by all authors and agrees to be responsible for all aspects of the work.

## Consent

Written informed consent was obtained from the son of the patient for publication of this case report and accompanying images. A copy of the written consent is available for review by the Editor-in-Chief of this journal on request.

## Registration of research studies

N/A.

## Guarantor

All co-authors bear full responsibility for the work and had access to the data, and controlled the decision to publish.

## Declaration of competing interest

No conflict of interest to declare.
